# First record of *Streptosyllis nunezi* Faulwetter et al., 2008 (Annelida, Syllidae) from the United Kingdom, and amendment to the genus *Streptosyllis* Webster & Benedict, 1884

**DOI:** 10.3897/zookeys.582.8006

**Published:** 2016-04-21

**Authors:** Will Musk, Sarah Faulwetter, Paul McIlwaine

**Affiliations:** 1 Institute of Estuarine and Coastal Studies, University of Hull, Cottingham Road, Hull, HU6 7RX, U.K.; 2 Institute for Marine Biology, Biotechnology and Aquaculture, Hellenic Centre for Marine Research, Thalassocosmos, Gournes, Heraklion, Crete, Greece; 3 The Centre for Environment, Fisheries and Aquaculture Science (Cefas), Pakefield Road, Suffolk, NR33 0HT, UK

**Keywords:** Polychaetes, simple ventral chaeta, generic amendment, identification key

## Abstract

During a benthic survey of a Marine Conservation Zone located on the Skerries Bank in the English Channel off the south-west coast of England, three specimens of *Streptosyllis nunezi* Faulwetter et al., 2008 were found. This is the second ever record of the species after its original description, and the first record from waters around the U.K. and a significant northerly range extension for a species previously only recorded from the Canary Islands and the Mediterranean Sea. A single simple ventral chaeta in each of the two posterior-most segments was discovered in this and two other species of *Streptosyllis* Webster & Benedict, 1884. The generic definition of *Streptosyllis* is emended to include this feature previously unknown for the genus, and an updated key to the *Streptosyllis* found in UK waters is provided.

## Introduction

Species of the genus *Streptosyllis* Webster & Benedict, 1884 are small-sized polychaetes living interstitially in shallow marine sediments of sand, muddy sand or sandy mud (San Martín 2003). The genus currently comprises 16 species (Gil and Read 2015) and is characterised by an unarmed pharynx, palps fused at the base, enlarged knob-tipped aciculae and modified compound chaetae in anterior segments (Brito et al. 2000, San Martín 2003). To date, five species of the genus have been reported from UK waters, or waters adjacent to the UK (Govaere et al. 1980, Howson and Picton 1997, San Martín and Worsfold 2015). Three of these species were originally described from Europe: *Streptosyllis bidentata* Southern, 1914 from Ballynakill, Ireland, *Streptosyllis campoyi* Brito, Núñez & San Martín, 2000 from the Canary Islands, Spain, and *Streptosyllis websteri* Southern, 1914 from Bofin, Ireland. The two other species, rarely reported from the UK, were originally described from the east coast of the USA: *Streptosyllis arenae* Webster & Benedict, 1884 from Provincetown, Massachusetts and *Streptosyllis varians* Webster & Benedict, 1887 from Eastport, Maine. The records for the latter two species however are questionable. Hartmann-Schröder (1996) considered the record of *Streptosyllis arenae* to be suspect, while Saint-Joseph’s (1895) description of *Streptosyllis varians* from Dinard (repeated in Fauvel 1923) does not fit the original description of Webster and Benedict (1887) and is believed to belong to a different species of the genus (Southern 1914).

In this paper, *Streptosyllis nunezi* is reported from UK waters for the first time and is the second record of the species after its original description. Furthermore, additional material of *Streptosyllis websteri* from both the UK and from Greece, and of *Streptosyllis campoyi* from the UK was examined and a previously unreported chaetal type was found in all three species of the genus. The generic definition of *Streptosyllis* is emended accordingly below.

## Methods

The Skerries Bank and Surrounds candidate Marine Conservation Zone (MCZ), situated off the south-west coast of England, was designated in November 2013 as part of the designation of 27 sites (Marine and Coastal Access Act 2009). In order to verify the species and habitats present, the Centre for Environment, Fisheries and Aquaculture Science (CEFAS) in collaboration with the Environment Agency (EA) undertook a benthic survey of the Skerries Bank and Surrounds site in January 2014. The benthos was sampled using a 0.1 m² Hamon grab, sieved through a 1.0 mm mesh and fixed in a buffered 4% formaldehyde seawater solution.

**Figure 1.**
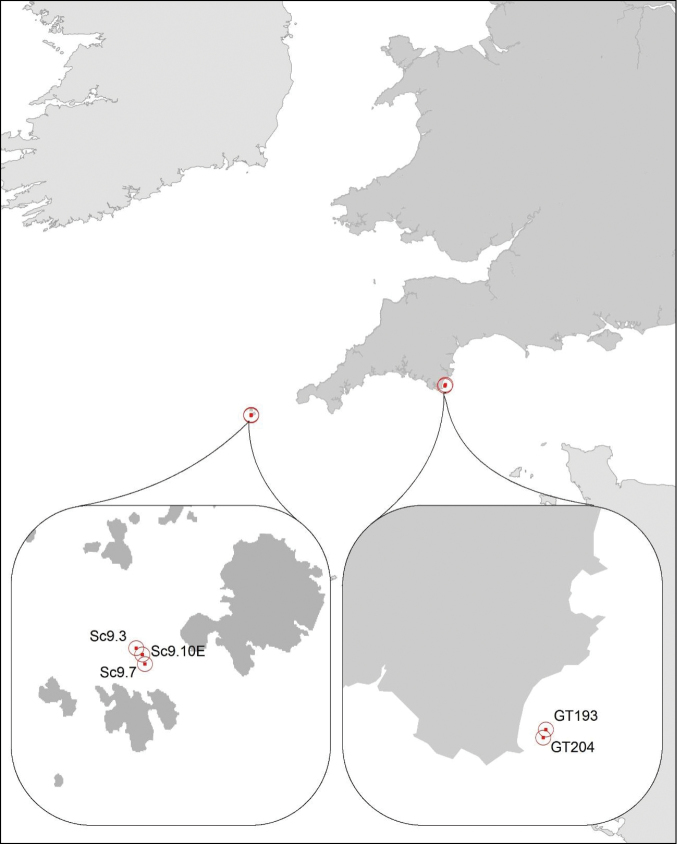
Location of sites off Devon and the Scilly Isles, UK, where *Streptosyllis nunezi* was recorded.

Specimens belonging to the genus *Streptosyllis* were found in the samples collected at sites GT193 (50.2537, -3.6058; depth 13.4 m) and GT204 (50.2429, -3.6110, depth 9.2 m) (Fig. 1). Both sites are located approximately 2.1 miles (3.5 km) from the coast, off Beesands in Devon, and are characterised by coarse to medium sands. The only other taxa common to both samples were *Nephtys cirrosa* Ehlers, 1868 and Nemertea species.

In addition, several *Streptosyllis* specimens in archive material of the Institute of Estuarine and Coastal Studies’ (IECS) were examined, which were collected during a benthic survey carried out by Natural England (NE) around the Scilly Isles in April 2009. *Streptosyllis* specimens were found at sites SC2.5 (49.9469, -6.2846; depth around 5–8 m), Sc9.3 (49.9078, -6.3436; depth 14.5 m), Sc9.7 (49.9042, -6.3401; depth 13.2 m) and Sc9.10E (49.9064, -6.3413; depth 11.8 m) from St. Mary’s Sound, Scilly Isles (Fig. 1), from coarse to medium sands. Samples were sieved through a 0.5 mm and 1.0 mm sieve; all specimens studied here were retained by the 0.5 mm fraction. Specimens were fixed and preserved in 70% Industrial Methylated Spirits (IMS). Samples were analysed in the laboratory by IECS, following national quality assurance guidelines for both faunal extraction and taxonomic identifications.

Additional specimens of *Streptosyllis campoyi* and *Streptosyllis websteri* from the IECS and of *Streptosyllis websteri* from archive collections of the Hellenic Centre for Marine Research (HCMR) were studied for comparison; sampling details of the latter can be found in Papageorgiou et al. (2006).

## Taxonomic results

During the identification phase of the analysis, three specimens of a *Streptosyllis* species with a distinctive hyaline hood on the blade and distal end of the shaft of the compound chaetae and serrated simple dorsal chaetae with a hyaline hood were found in samples GT193 and GT204. A further six individuals with the same characteristics were discovered in the IECS archive material in samples Sc9.3, Sc9.7 and Sc9.10E. The chaetae found in the specimens were unlike those of any of the *Streptosyllis* species confirmed so far from UK waters: *Streptosyllis websteri* Southern, 1914, *Streptosyllis bidentata* Southern, 1914 and *Streptosyllis campoyi* Brito, Núñez & San Martín, 2000 (San Martín and Worsfold 2015). After comparison with the literature and personal notes of the second author, the specimens were confirmed to belong to *Streptosyllis nunezi*, a species so far only known from Crete, Tuscany and the Canary Islands. The examination of the specimen also revealed a single simple ventral chaeta in each parapod of the last two fully formed posterior chaetigers, a character previously thought to be absent in the genus *Streptosyllis* (San Martín 2003, San Martín and Hutchings 2006). Upon examination, specimens of *Streptosyllis campoyi* and *Streptosyllis websteri* from the IECS reference collection and archived material, as well as archived material of *Streptosyllis websteri* from Crete were found to also possess a simple ventral chaetae in the last one or two fully formed posterior chaetigers. Based on this newly discovered character, the generic diagnosis for *Streptosyllis* is emended below.

### Genus Streptosyllis

Taxon classificationAnimaliaPhyllodocidaSyllidae

Webster & Benedict, 1884, emended

Streptosyllis Webster & Benedict, 1884: 711
Streptosyllis – San Martín 2003: 120
Streptosyllis – San Martín and Hutchings 2006: 354
Streptosyllis – Faulwetter et al. 2008: 2

#### Type species.

*Streptosyllis arenae* Webster & Benedict, 1884.

#### Diagnosis.

Body small. Four eyes, occasionally anterior pair of eyespots present. Palps fused at base, occasionally reduced to small papillae. Anterior parapodia with modified compound chaetae; sometimes with enlarged aciculae. Dorsal simple chaetae present, simple ventral capillary chaetae may be present in posteriormost chaetigers. Compound chaetae homogomph or hemigomph, falcigerous or spinigerous. Dorsal cirri smooth, pseudoarticulated or articulated with granular inclusions. Ventral cirri digitiform, sometimes longer than parapodial lobe. Pharynx unarmed with crown of soft papillae. Pygidium with one median and two lateral anal cirri.

### Streptosyllis nunezi

Taxon classificationAnimaliaPhyllodocidaSyllidae

Faulwetter, Vasileidadou, Papageorgiou & Arvanitidis, 2008

Streptosyllis nunezi Faulwetter, Vasileidadou, Papageorgiou & Arvanitidis, 2008: 5, figs 4–6.

#### Material examined.

3 individuals from the Skerries Bank, England, 9–13 m depth; 6 individuals from the Scilly Isles, England, 11–14 m depth, at both sites in coarse to medium sand.

#### Description.

**Figure 2.**
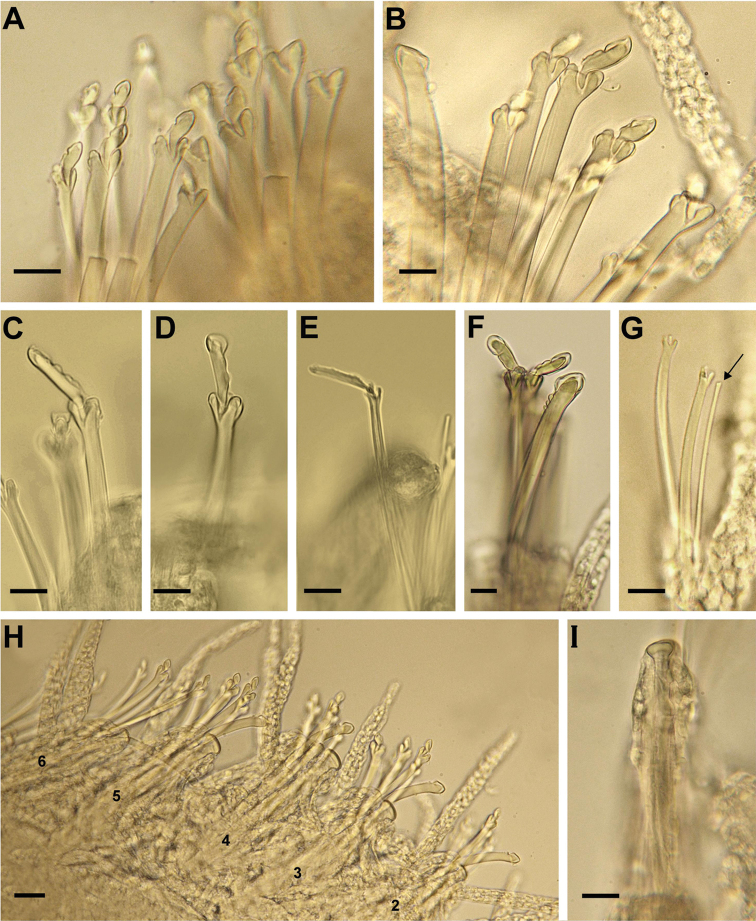
*Streptosyllis nunezi*, **A** anterior parapodium with falcigers with short blades **B** parapodium, mid-body **C, D** falcigers with elongated blade, mid-body; **E** falciger with elongated blade, posterior chaetiger **F** dorsal simple chaeta **G** posterior parapodium with ventral simple chaeta (arrow) **H** aciculae of chaetigers 2–6 (numbered) **I** acicula, mid-body. Scale bars: 10 µm (**A–G, I**), 20 µm (**H**).

Body ca. 5 mm long, for 64 chaetigers in the only complete animal. Head semi-circular with two pairs of eyes and two eyespots located anteriorly. Three smooth antennae, median one twice as long as lateral ones. Palps basally fused, forming two rounded lobes, not visible dorsally. Two pairs of smooth tentacular cirri, about as long as lateral antennae. Dorsal cirri about as long as or slightly shorter than body width, smooth anteriorly, after proventricular region at irregular distances with pseudo-articulations containing yellow granular inclusions. Ventral cirri digitiform, smooth, almost as long as parapodial lobes anteriorly and in midbody, longer than parapodial lobe posteriorly. Posteriormost 3 segments achaetous. Pygidium with single filiform ventral cirrus (2 lateral cirri missing?). Up to 8 compound falcigers in each parapodium. Shafts of compound chaetae with three hemigomph teeth, sometimes notched so that they appear as up to four teeth (Fig. 2A–B). Blades of falcigers of two types: short ones (ca. 7–9 µm) and longer ones (ca. 15 µm), former ones occurring in anteriormost chaetigers, longer ones in two dorsalmost chaetae of midbody and in posterior chaetigers (Fig. 2C–E). Short blades covered entirely by membrane forming blunt tip and notch alongside of blade; longer blades covered by membrane forming pointed tip if viewed laterally, blunt if viewed from top, and 1–2 teeth along cutting edge of blade (Fig. 2C–D). Membrane of blades often extending to shaft, covering its top. Posteriorly, all blades of compound chaetae thin and elongated (Fig. 2E). One dorsal simple chaeta present per chaetiger, from anteriormost chaetigers, slightly curved, tip bluntly rounded, covered by membrane forming blunt tip. Strong serration on distal end just below hood, forming up to 4 large, round teeth (Fig. 2F). One ventral simple chaeta in each of two last posteriormost chaetigers (excluding developing ones), very thin, capillary-like (Fig. 2G). Single acicula per parapodium, distally knobbed, knob sometimes irregularly rounded with one side longer than the other, anteriorly sometimes protruding from parapodium. Aciculae slightly enlarged in segments 3, 4, and 5, about 1.5–2 times larger than those of preceding and proceeding chaetigers (Fig. 2H–I). Pharynx through 3–5 segments, proventricle through 6 segments.

#### Remarks.

Except for the presence of ventral chaetae and the aciculae protruding from the parapodium, all examined animals correspond well to the original description by Faulwetter et al. (2008).

#### Distribution.

Mediterranean Sea (Crete, Italy), northeastern Atlantic (Canary Islands, Scilly Islands, Skerries Bank)

#### Ecology.

Occurs in fine to coarse sandy substrates in shallow waters (1–20 m).

### Streptosyllis campoyi

Taxon classificationAnimaliaPhyllodocidaSyllidae

Brito, Núñez & San Martín, 2000

Streptosyllis campoyi Brito, Núñez & San Martín, 2000: 611, figs 5a–l.
Streptosyllis bidentata (non Southern) – Campoy 1982: 314, figs 25 a–j.
Streptosyllis campoyi – San Martín 2003: 131, figs 63–64.

#### Material examined.

3 individuals from Station Sc9.7, St. Mary’s Sound, Scilly Isles, 13.2 m depth, in coarse to medium sand.

#### Remarks.

**Figure 3.**
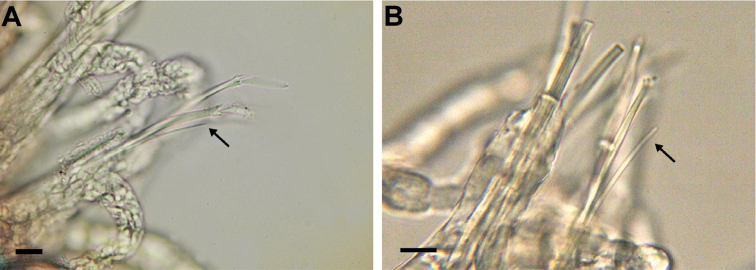
Posterior parapodium with ventral simple chaeta (arrow) of **A** *Streptosyllis campoyi* **B** *Streptosyllis websteri*. Scale bars: 10 µm

All examined animals correspond well to the description provided by San Martín (2003), except for the presence of a thin, capillary-like ventral simple chaeta in each of the two posteriormost chaetigers (Fig. 3A).

### Streptosyllis websteri

Taxon classificationAnimaliaPhyllodocidaSyllidae

Southern, 1914

Streptosyllis websteri Southern, 1914: 26, pl. II, figs 3 a–f.
Streptosyllis websteri – Hartmann-Schröder 1996: 163, fig. 69; San Martín 2003: 125, figs 59–60; San Martín and Aguado 2006: 731, fig. 2; Nygren and Pleijel 2015: 182.

#### Material examined.

15 individuals from Elafonisi Island, Crete, 0–1 m depth; 1 individual from Pahia Ammos, Crete (5 m depth), at both sites in coarse sand; 2 individuals from Station Sc2.5 Scilly Isles, depth around 5–8 m.

#### Remarks.

All examined animals correspond well to the description provided by San Martín (2003), except for the presence of a single thin, capillary-like ventral simple chaeta in each of the two posteriormost chaetigers (Fig. 3B).

## Discussion

*Streptosyllis arenae* is morphologically the most similar species to *Streptosyllis nunezi*; it differs from *Streptosyllis arenae* by the presence of the hyaline hood covering the distal end of the shaft of the compound chaetae, and the rounded teeth found on the shaft of the dorsal simple chaetae. Other *Streptosyllis* species which have a hyaline hood on the blades of their compound chaetae are *Streptosyllis biarticulata* Hartmann-Schröder, 1991, and *Streptosyllis magnapalpa* Hartmann-Schröder, 1981. *Streptosyllis nunezi* can also be distinguished from both these species again by the presence of the hyaline hood covering the distal end of the shaft of the compound chaetae, and the rounded teeth found on the shaft of the dorsal simple chaetae (Faulwetter et al. 2008); furthermore these species are only known so far from Australia. *Streptospinigera templadoi* (San Martín, 1984) also has hyaline hood- like structures on its compound chaetae, however it also lacks the hyaline hood on the distal end of the shaft of the compound chaetae and teeth on the dorsal simple chaetae, furthermore it possesses spinigerous compound chaetae not seen in *Streptosyllis nunezi* (Olivier et al. 2013).

*Streptosyllis arenae* was recorded by Govaere et al. (1980) and Vanosmael et al. (1982) from the southern North Sea, but whether these records really pertain to *Streptosyllis arenae*, originally described from Provincetown, Massachusetts, USA, is unknown, though Hartmann-Schröder (1996) suspected them to be *Streptosyllis websteri*. These records might in fact also belong to *Streptosyllis nunezi*, which is here shown to have a more northerly distribution than previously known, but investigation of the material would be needed to confirm this. Recently, *Streptosyllis nunezi* has been recorded by one of us (WM) from the eastern Humber region of the North Sea. This could lend additional support to the hypothesis that previous records of *Streptosyllis arenae* from the North Sea could be referred to *Streptosyllis nunezi* and that the species might be native to the region. Its small size and the fact that only recently appropriate keys for the group have been published might have contributed to it being overlooked or misidentified in the past. An updated key to the UK species of the genus is provided below.

### Key to the *Streptosyllis* (Webster & Benedict, 1884) species found in UK waters

(adapted from San Martín and Worsfold (2015))

**Table:** 

1	Compound chaetae with hyaline hood-like structures around the blade and distal end of the shaft and a strong serration on the distal end of the simple dorsal chaetae	***Streptosyllis nunezi*Faulwetter et al., 2008**
–	Compound chaetae without hyaline hood-like structures	**2**
2	Compound chaetae with indistinctly bidentate blades. Strongly enlarged aciculae in chaetigers 2–5	***Streptosyllis websteri* Southern, 1914**
–	Compound chaetae with distinctly bidentate blades. Strongly enlarged aciculae in chaetigers 2–6	**3**
3	Blades of compound chaetae with both teeth similar and close to each other. Aciculae of chaetiger 7 only slightly more slender than those of chaetiger 6	***Streptosyllis bidentata* Southern, 1914**
–	Blades of compound chaetae with proximal teeth longer and well separated. Aciculae of chaetiger 7 distinctly more slender than those of chaetiger 6	***Streptosyllis campoyi* Brito, Núñez & San Martín, 2000**

## Conclusions

In conclusion, the findings of this study showed a significant northerly range extension for *Streptosyllis nunezi* previously only confirmed from the Canary Islands and the Mediterranean Sea. A single simple ventral chaetae in each of the two posteriormost segments was also discovered in this and two other species of *Streptosyllis*, resulting in an emended diagnosis of the genus.

## Supplementary Material

XML Treatment for Streptosyllis

XML Treatment for Streptosyllis nunezi

XML Treatment for Streptosyllis campoyi

XML Treatment for Streptosyllis websteri

## References

[B1] Brito MC Núñez J San Martín G (2000) The genus *Streptosyllis* Webster and Benedict, 1884 (Polychaeta: Syllidae: Eusyllinae) from the Canary Islands, with description of a new species. Bulletin of Marine Science 67: 603–615. http://www.ingentaconnect.com/content/umrsmas/bullmar/2000/00000067/00000001/art00048

[B2] Campoy A (1982) Fauna de España de Anélidos Poliquetos de la Península Ibérica. Publicaciones de Biología de la Universidad de Navarra, Serie Zoología 7: 1–463. http://hdl.handle.net/10171/11773

[B3] Ehlers E (1868) Die Borstenwürmer (AnnelidaChaetopoda) nach systematischen und anatomischen Untersuchungen dargestellt. Wilhelm Engelmann Verlag, Leipzig, 748 pp. doi: 10.5962/bhl.title.2081

[B4] Faulwetter S Vasileiadou A Papageorgiou N Arvanitidis C (2008) Description of a new species of *Streptosyllis* (Polychaeta: Syllidae) from the Mediterranean and Canary Islands with a re-description of *Streptosyllis arenae* and comments on the taxonomy of *Streptosyllis* and some morphologically similar genera. Zootaxa 1847: 1–18. http://www.mapress.com/zootaxa/2008/f/zt01847p018.pdf

[B5] Fauvel P (1923) Polychètes Errantes. Faune de France, Paris, 488 pp http://www.faunedefrance.org/bibliotheque/docs/P.FAUVEL(FdeFr05)Polychetes-errantes.pdf

[B6] Gil J Read G (2015) *Streptosyllis* Webster and Benedict, 1884. In: Read G Fauchald K (Eds) World Polychaeta database. Accessed through: http://www.marinespecies.org/aphia.php?p=taxdetailsandid=129678 [2016-01-20]

[B7] Govaere JCR Van Damme D Heip C De Coninck LAP (1980) Benthic communities in the Southern Bight of the North Sea and their use in ecological monitoring. Helgoländer Meeresuntersuchungen 33: 507–521. doi: 10.1007/BF02414775

[B8] Hartmann-Schröder G (1981) Die Polychaeten der tropisch-subtropischen Westküste Australiens (zwischen Exmouth im Norden und Cervantes im Süden). Mitteilungen aus dem Hamburgischen Zoologischen Museum und Institut 78: 19–96.

[B9] Hartmann-Schröder G (1991) Die Polychaeten der subtropisch-tropischen bis tropischen Ostküste Australiens zwischen Maclean (New South Wales) und Gladstone (Queensland) sowie von Heron Island (Großes Barriere-Riff). Mitteilungen aus dem Hamburgischen Zoologischen Museum und Institut 88: 17–71.

[B10] Hartmann-Schröder G (1996) Annelida, Borstenwürmer, Polychaeta. 2., neubearbeitete Auflage. Gustav Fischer Verlag, Jena, 648 pp.

[B11] Howson CM Picton BE (1997) The species directory of the marine fauna and flora of the British Isles and surrounding areas. Marine Conservation Society and Ulster Museum, Ross-on Wye and Belfast, 508 pp.

[B12] Marine and Coastal Access Act (2009) Marine and Coastal Access Act. http://www.legislation.gov.uk/ukpga/2009/23/contents

[B13] Nygren A Pleijel F (2015) Ringmaskar: Havsborstmaskar, Annelida: Polychaeta. Art-Databangken, SLU, Uppsala, 346 pp http://www.artdatabanken.se/media/2291/lagupp_havsborstmaskar.pdf

[B14] Olivier F San Martín G Archambault P (2013) A new species of *Streptospinigera* Kudenov, 1983 (Polychaeta, Syllidae, Anoplosyllinae) from the Arctic and north-western Atlantic with a key to all species of the genus. Polar Biology 36: 1499–1507. doi: 10.1007/s00300-013-1369-6

[B15] Papageorgiou N Arvanitidis C Eleftheriou A (2006) Multicausal environmental severity: A flexible framework for microtidal sandy beaches and the role of polychaetes as an indicator taxon. Estuarine, Coastal and Shelf Science 70: 643–653. doi: 10.1016/j.ecss.2005.11.033

[B16] Saint-Joseph A (1895) Les Annélides polychètes des côtes de Dinard, pt. 4. Annales des sciences naturelles Ser. 7 20: 185–272. http://biodiversitylibrary.org/page/35660882

[B17] San Martín G (1984) Estudio biogeográfico, faunístico y sistemático de los poliquetos de la familia Sílidos (Syllidae: Polychaeta) en Baleares. PhD Thesis, Universidad Complutense de Madrid, Madrid, 529 pp.

[B18] San Martín G (2003) Annelida, Polychaeta II: Syllidae. In: Ramos MA et al. (Eds) Fauna Ibérica (Vol. 21). Museo Nacional de Ciencias Naturales, CSIC, Madrid, 554 pp.

[B19] San Martín G Aguado MT (2006) Sílidos (Syllidae: Polychaeta) del Parque Nacional de Coiba (Pacífico, Panamá). Revista de Biologia Tropical 54: 725–743. 18491613

[B20] San Martín G Hutchings PA (2006) Eusyllinae (Polychaeta: Syllidae) from Australia with the description of a new genus and fifteen new species. Records of the Australian Museum 58: 257–370. doi: 10.3853/j.0067-1975.58.2006.1466

[B21] San Martín G Worsfold TM (2015) Guide and keys for the identification of Syllidae (Annelida, Phyllodocida) from the British Isles (reported and expected species). ZooKeys 488: 1–29. doi: 10.3897/zookeys.488.9061 25878521 10.3897/zookeys.488.9061 PMC4389122

[B22] Southern R (1914) Clare Island Survey. Archiannelida and Polychaeta. Proceedings of the Royal Irish Academy 31: 1–160. http://biodiversitylibrary.org/page/34773787

[B23] Vanosmael C Willems KA Claeys D Vincx M Heip C (1982) Macrobenthos of a Sublittoral Sandbank in the Southern Bight of the North Sea. Journal of the Marine Biological Association of the United Kingdom 62: 521–534. doi: 10.1017/S002531540001972X

[B24] Webster HE Benedict JE (1884) The Annelida Chaetopoda from Provincetown and Wellfleet, Massachusetts. Annual Report of the United States Commission of Fish and Fisheries, Washington 1881: 699–747. http://biodiversitylibrary.org/page/11203280

[B25] Webster HE Benedict JE (1887) The Annelida Chaetopoda, from Eastport, Maine. Annual Report of the United States Commission of Fish and Fisheries, Washington 1885: 707–758. http://biodiversitylibrary.org/page/15839855

